# Supplementary material for: Barriers to integration of bioinformatics into undergraduate life sciences education: A national study of US life sciences faculty uncover significant barriers to integrating bioinformatics into undergraduate instruction
Source: PLoS One. 2019 Nov 18;14(11):e0224288. doi: 10.1371/journal.pone.0224288 (PMC6860448; doi:10.1371/journal.pone.0224288)
Supplement: S1 Responses — (PDF) [file pone.0224288.s001.pdf]

To illustrate the most prominent barriers, we include relevant quotes from survey respondents below. They have been lightly edited for clarity and to correct typographical errors.

Super-category: Faculty issues. Category: Lack of expertise/training

> "Lack of training in the area, as many of us earned our PhDs before bioinformatics was widely used and available."

> "Lack of faculty expertise in the rapidly changing disciplines of genomics/bioinformatics and related technologies."

> "A lack of training and experience in bioinformatics."

Super-category: Faculty issues. Category: Lack of time

> "A lack of agreement about what topics should be dropped in order to have space/time to introduce bioinformatics."

> "Time to fit into the curriculum."

> "Too much other stuff in the Biology curriculum that is perceived as essential."

Super-category: Student issues. Category: Lack of background skills/knowledge

> "The lack of basic mathematical and computational knowledge and skills among students, even in a private university."

> "The students' ability to think critically and effectively evaluate information. They rely on being told exactly what to do and have an inability to problem solve when things don't turn out as they anticipate."

> "Biology [students] shy away from mathematical and statistical methods, and few have programming experience."

Super-category: Student issues. Category: Lack of interest

> "Finding ways to make students interested and engaged in the material presented."

> "Student skepticism/resistance."

> "Apathy on the part of students."
