# Supplementary material for: Barriers to integration of bioinformatics into undergraduate life sciences education: A national study of US life sciences faculty uncover significant barriers to integrating bioinformatics into undergraduate instruction
Source: PLoS One. 2019 Nov 18;14(11):e0224288. doi: 10.1371/journal.pone.0224288 (PMC6860448; doi:10.1371/journal.pone.0224288)
Supplement: Attachment — Submitted filename: Response to Reviewers.pdf [file pone.0224288.s002.pdf]

National Science Foundation
Directorate for Education and Human Resources

28 September 2019

Dear Dr. Bianchi,

I would like to thank the reviewers of our paper for their helpful comments and kind words. The other authors and I have addressed their concerns in the attached versions of the manuscript. Below I give the concerns themselves and our responses to them. (Section headers from the review are in **bold**, reviewers' comments are in *italics*, and our responses are in plain type.)

Note that not tracked in the attached is a modification to the author list the paper: due to a change in their last name, one of the authors was moved to a different place in the list.

3. Have the authors made all data underlying the findings in their manuscript fully available?

Reviewer #1 answered "No" to this question. However, as stated on Line 586 of the original manuscript (now Line 595), all our data and analyses are available on our GitHub repository (https://github.com/niblse).

5. Review Comments to the Author

Reviewer #1: I have some concerns with inconsistencies in the way the methodology is presented in the paper.

In the abstract, the mode of analysis that was used to identify barriers was not mentioned. The writing of the abstract suggests the barriers were identified quantitatively. It is important to make clear in the abstract that this is a mixed methods study that uses qualitative analysis.

To address this comment, we have added the following sentence to the abstract: "The survey asked several open-ended questions that probed barriers to integration the answers to which were analyzed using a mixed-methods approach." (Line 104).

It is not clear from the methods description whether the keyword analysis was inductive or deductive i.e. were the keywords and supercategories pre-determined or did they arise from the responses themselves. This is a very important methodological question. Either is fine.

The analysis was deductive—the categories and super-categories arose from the responses themselves. To make this clear, the word "deductive" has been added to the descriptions in the Introduction (Line 139), Results (Line 165), and Materials and Methods (Line 555) sections.

In the introduction (line 136) the analysis of free response questions is described as keyword analysis. In line 177 in results its again described as keyword analysis. Elsewhere in line 175 and 542 its described as qualitative analysis. These are minor distinctions, but since many of the readers will be from the life sciences and have limited knowledge of qualitative approaches - clarity is very important.

We have changed the descriptions of this to "qualitatively analyzed for specific barriers that deductively arose" (or similar) (Lines 139, 164, and 180).

Similarly, the methods section has some missing citations:
line 543 citation missing for Leech and Onweugbuzie
line 545 citation missing for Harding

Thank you for pointing this out. For some reason, these citations were "hidden" in the submitted version of the manuscript. They are now unhidden.

The manuscript also does not include a supplement that shows de-identified example responses that align with each code.

We have added a supplement with example responses.

Similarly, the manuscript does not describe any plans for releasing the data, or provide reasons why the data will not be released.

As described above, our data and analyses are available on GitHub.

Reviewer #2: This work addresses an important problem: bioinformatics training. It has done this through a standard survey method. The methodology seems sound to me, and the level of responses obtained seems what one would expect from such a survey. Of course there are limitations when the level of response is 1 or 2% of the population. With that as a caveat, I would say that the results of this survey are likely representative of the intended population.

*The main result is that even in 2019 bioinformatics still faces training barriers. This matches my individual experience. Even though in that sense the result is not surprising, taken in perspective (25 years since the H. influenzae genome was published) it *is* surprising. Given the importance of the field, 20 years ago I had the expectation that it would have been much easier to adopt bioinformatics in curricula by now, and this, sadly, is not yet the case. So I think this work will be an important contribution, which will help show university officials that there is a real need to help facilitate the introduction of bioinformatics in curricula.*

Minor comments:
1) there are several empty references in Methods. Eg. "...at the 95% confidence interval []".

As noted above, these references are now visible.

2) I think the authors should cite (and comment) this report:
A New Biology for the 21st Century
Committee on a New Biology for the 21st Century:
Ensuring the United States Leads the Coming Biology
Revolution; National Research Council, 2009

This is an important document, which makes several specific recommendations. Many of them are related to the development of quantitative skills and interdisciplinary education. Bioinformatics is of course of paramount importance in both. By the way, it is curious that this manuscript does not use the word "interdisciplinary" even once.

Thank you for bringing this reference to our attention. We agree it is important and have concluded the Discussion with a citation to it (Line 527).

In addition, we have pointed out the interdisciplinary nature of bioinformatics in Lines 115, 134, and 433.

3) the definition given for bioinformatics is "a discipline that combines aspects of biology, statistics, and computer science". Math is left out, and some mathematicians who see themselves as contributing to bioinformatics may take offence. The authors may wish to use a more generic term, such as "exact sciences" or "quantitative sciences" to avoid this problem.

We have added the word "mathematics" to the list of disciplines that bioinformatics draws from (Lines 99 and 116).

4) I wish the authors would have gone a bit further in their discussion, and have made comments on the more general problem of barriers to any interdisciplinary education. In my view, despite all the rethoric one hears about the importance of interdisciplinary research and education, at the undergraduate classroom level we still suffer from the old discipline divisions (departmental silos certain are a major factor). One would think that this could have been a generational issue, but one of the findings of this work (that recently graduated faculty are the least likely to integrate bioinformatics into their teaching) suggests that it's not; which means that something needs to be done, besides waiting old monodiscipline diehards to retire.

Yes, we agree. We have expanded on this idea starting in Line 432 of the Discussion.

Thank you again for your consideration of the manuscript. If you have any questions about the changes that were made, please feel free to contact me by phone (703-292-8658) or e-mail (*mpauley@nsf.gov*).

Sincerely,

Mark A. Pauley, Ph.D.
Program Director
Division of Undergraduate Education
